# New Method for Photoactive Cement Preparation—Selected Mechanical Properties and Photocatalytic Activity of New Materials

**DOI:** 10.3390/ma17102285

**Published:** 2024-05-11

**Authors:** Magdalena Janus, Jarosław Strzałkowski, Kamila Zając, Ewelina Kusiak-Nejman

**Affiliations:** 1 Faculty of Civil and Environmental Engineering, West Pomeranian University of Technology in Szczecin, al. Piastów 50, 70-311 Szczecin, Poland; magdalena.janus@zut.edu.pl (M.J.); jaroslaw.strzalkowski@zut.edu.pl (J.S.); kamila.zajac@zut.edu.pl (K.Z.); 2 Department of Inorganic Chemical Technology and Environment Engineering, Faculty of Chemical Technology and Engineering, West Pomeranian University of Technology in Szczecin, ul. Pułaskiego 10, 70-310 Szczecin, Poland

**Keywords:** photocatalysis, photoactive cement, NO decomposition

## Abstract

In this study, a new method of obtaining photoactive cements is presented. The goal was to obtain photoactive cements using a method that could reduce the production costs. In the study, an intermediate product from the production of titanium dioxide using the sulfate method, taken from the installation before the calcination process, was used to obtain photoactive cements. Laboratory conditions corresponding to introducing this amorphous TiO_2_ into cement clinker during its cooling were simulated. The study shows that the temperature from 300 to 800 °C and the time of amorphous TiO_2_ contact with the cement clinker within 30 min is sufficient to obtain a photoactive cement. The highest photocatalytic activity was obtained for the material with 5 wt.% TiO_2_ content, and the method used did not cause a significant decrease in the bending and compressive strength of the new photoactive cements. The obtained materials were characterized by determining the crystal size of the TiO_2_, the sulfur content and the photocatalytic activity during NO decomposition under UV radiation. The bending and compressive strength were measured. The influence of the addition of photocatalysts on the beginning and end of the setting time was also investigated.

## 1. Introduction

Photocatalysis has aroused great interest in recent years, as it is called reverse photosynthesis. Sunlight is needed to initiate both processes. As a result of photosynthesis, the pigment in the leaves can convert carbon dioxide and water into organic compounds and oxygen under the influence of radiation. Photocatalysis is reverse photosynthesis because the opposite occurs on the photocatalysts’ surface. Under the influence of light, several reactions take place on the surface of photocatalysts, which lead to the production of several reactions on the surface of photocatalysts, which produce hydroxyl radicals, which can decompose organic compounds into carbon dioxide and water. Firstly the photocatalyst particles absorb a photon of energy equal to or greater than a band gap energy (Eg) of the semiconductor. Electrons are transferred from the conduction band to the valance band, and electron–hole pairs (e^−^–h^+^) appear. After that, reactive oxygen species (e.g., ^•^OH, HO_2_^•^, O_2_^•−^) are formed; these species take part in pollution decomposition. The photocatalytic process depends on: (1) the absorption of photons by the photocatalyst; (2) the adsorption of pollutant molecules on the photocatalyst surface; (3) the quick charge separation; (4) the low charge recombination. Nowadays, it would be difficult to imagine modern construction and architecture without concrete, a widely used construction material. However, it is not only the functionality, aesthetics and durability of buildings that justify using this composite material. Currently, an important argument in favor of its use is the contribution to protecting the natural environment [1,2]. TiO_2_ is a photocatalyst that is often added to various types of building materials, such as glass, cement, gypsum and others, in order to give them self-cleaning properties, as well as to make them able to clean the air from pollutants [3,4]. We can distinguish three main methods of obtaining photoactive building materials. One of them is incorporating TiO_2_ into the total weight of building materials such as cement or gypsum. The second method is covering the building material with a thin layer of photoactive materials, e.g., paint or a thin layer of photocatalyst. The third method is covering the building materials with thicker layer of photoactive materials, e.g., photoactive plaster on other building materials. The first modification involves the addition of different amounts of TiO_2_ concerning the total weight of the cement (cements modified in bulk with TiO_2_) in the form of a photocatalyst suspension or dry powder and requires only a simple mixing of the two raw materials [5,6]. It is well known that heterogeneous photocatalysis is a process that occurs only on the surface of a material. Therefore, utilizing the method of incorporating the photocatalyst in the whole mass limits the photocatalytic efficiency of such material because the sun’s rays do not reach the deeper layers of cement [7]. On the other hand, such modification could improving the mechanical properties of cements by additional filling of pores and the interaction of TiO_2_ with cement components [5]. According to Wang et al. [8], the use of surface treatment technology reduces costs due to the lower need for a photocatalyst. In addition, compared to the built-in method, surface treatment technology using photocatalysts has a wider scope, as it is possible to impart photocatalytic properties to existing buildings. However, the solution of enriching the surface of the cement mortar is limited by the poor adhesion of the photocatalytic material to the substrate [9]. Hernández-Rodríguez et al. [10] imparted photoactive properties by incorporating TiO_2_ into only a one-centimeter surface layer of cement mortar samples.

Cement is produced by two methods: dry and wet. Currently, the dry method of obtaining cement is dominant in the world. This method involves introducing cement flour into a kiln to produce clinker. In this method, the clinker is calcinated at 1450–1500 °C to achieve the required level of combination; after that, the hot clinker is passed through a cooler [11]. The clinker cooling process is very fast. The temperature of the clinker is about 100 °C in the cooler within 30 min.

The knowledge about the temperature in the cooler was used to carry out research to check whether the time of 30 min and the temperature in the cooler is sufficient to obtain photoactive cement, if the intermediate product from the TiO_2_ production plant using the sulfate method is introduced to the appropriate place in the cooler and whether 30 min is sufficient for the amorphous form of TiO_2_ to transform into photoactive anatase. In this article, we would like to present studies about a new and cheaper method for photoactive cement preparation.

## 2. Materials and Methods

### 2.1. Obtaining Modified Cements

The cement clinker (Lafarge, Piechnin, Poland) was annealed at 300, 600 and 800 °C. Then, during the cooling process, which lasted 30 min, an intermediate product from the TiO_2_ production plant by the sulfate method (called amorphous TiO_2_) was added to the clinker in amounts of 1, 3 and 5 wt.% of the clinker weight on a dry matter basis. The semi-finished product from the installation of TiO_2_ production was taken from drum filters before the addition of roasting additives and before calcination. This material contained 60% water. During cooling, the clinker was mixed with an appropriate amount of TiO_2_, and then it was ground with 5 wt.% gypsum obtained from the flue gas desulfurization installation of the Dolna Odra power plant (Nowe Czarnowo, Poland) for 35 min at a speed of 450 rpm. Designations of materials adopted in the studies in Table 1 was presented.

### 2.2. Sample Preparation for XRD and Sulfur Content Analysis

In order to check how TiO_2_ behaves when it contacts with clinker at different temperatures during 30 min of cooling, a process simulating these conditions was carried out. Ceramic spheres were used instead of clinker because, thanks to this, it was possible to measure the size of crystallites and sulfur content without clinker, which interfered with the measurement. The ceramic balls with a diameter of 1 cm were annealed at 300, 600 and 800 °C. Then, during the cooling process, which takes 30 min, amorphous TiO_2_ was added to the ceramic balls in amounts of 1, 3 and 5 wt.% of the weight of the ceramic balls on a dry matter basis.

The crystal structure of investigated samples was characterized by XRD analysis (Empyrean X-ray diffractometer, PANalytical, Almelo, The Netherlands) using Cu Kα radiation with a wavelength of 1.54056 Å. Diffractograms were conducted in the 2θ angular range from 20 to 80°. The average crystallite size was calculated based on the Rietveld method.

A CS230 elemental analyzer (LECO Corporation, St. Joseph, MI, USA) was utilized to determine the total sulfur content in the tested titania-based samples. A certified BCS-CRM No. 362 mine tailings sample (Bureau of Analysed Samples Ltd., Newham Hall, Middlesbrough, UK) containing 1.4848 wt.% of sulfur was used to prepare the calibration curve. The calculated value is the average value of at least two measurements. Values were excluded from the average if the observational error exceeded 5%.

### 2.3. The Beginning and End of the Setting Time Measurements

Before determining the beginning and end of the setting of individual grouts, the standard consistency of the slurry was determined with the Clinker sample + 5% gypsum. The standard distance between the pin and the base plate reached a height of 6 ± 2 mm with 152.0 g of water per 500.0 g of cement, and the W/C was ~0.30. The value of W/C = 0.30 was applied to all other variants of the tested grouts. The tests were carried out using Vicata Vicatronic automatic devices (Matest company, Treviolo, Italy). For each slurry, two measurements of the beginning and end of the setting time were taken, which were then averaged. The tests were carried out following the standard PN-EN 196-3:2016 [12]. The samples were stored under water at a temperature of 20 ± 1 °C. Time intervals of 5 min between each reading were assumed. The time after the needle stopped at a height of 6 ± 3 mm from the base plate was recorded as the beginning of the setting time. On the other hand, the end of the setting time corresponded to the needle depression to a depth of 0.5 mm from the top surface of the sample.

### 2.4. Determination of Bending and Compressive Strength of Standard Mortar

The strength test was carried out based on the standard PN-EN 196-1:2016 [13] and was carried out on 4 × 4 × 16 cm beams made of mortar with a standard composition: 450 g of cement, 1350 g of standard sand and 225 g of water. The W/C ratio was assumed to be constant and was 0.5 in all mortars. After the samples, they were stored in a water bath with increased humidity. After 24 h, the samples were demolded and stored underwater until they were tested. The tests were performed after 28 days of puberty. Each time, the bending strength measurements Rf were performed on three beams, and the measurements of compressive strength Rc were performed on six specimens. The tests were carried out on standard Walter + Bai equipment for strength measurements. In addition to mean values, standard deviations and coefficients of variation were also determined.

### 2.5. Photocatalytic Activity of the Investigated Materials

**Figure 1:**
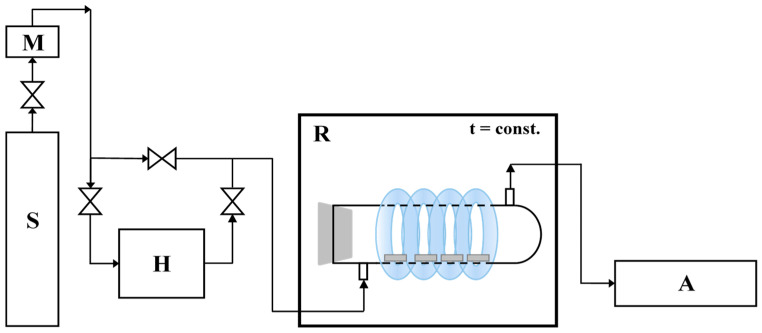
The scheme of installation to photocatalytic removal of NO (S—a source of pollution; M—mass flower; H—humidifier; R—photocatalytic reactor with irradiation source; A—NOx analyzer [3].

In our previous work [3], the NO gas (1.989 ppm ± 0.040 ppm, Air Liquide, Kraków, Poland) was used as model pollution in photocatalytic tests. NOx removal was evaluated using the experimental installation, a scheme of which is presented in Figure 1.

Eight studied cement plates (one at dimensions of 2 × 2 × 0.5 cm) were placed in the central part of the cylindrical reactor (Pyrex glass; Ø × H = 9 × 32 cm), and the reactor was tightly closed. The NO was diluted with humidified synthetic air in a ratio of 1:1 to obtain 1.25 mg·m^−3^ initial concentration. The NO was diluted with synthetic to obtain a 0.2 mg·m^−3^ initial concentration. The oxygen and water molecules were necessary for forming oxidative species, which are essential in photocatalytic reactions. The polluted air flowed through the reactor continuously at a rate of 500 cm^3^·min^−1^. The dark conditions were maintained at the beginning of the process until NO concentration reached equilibrium (about 1.25 mg·m^−3^ for about 35 min). Then, the UV lamps were turned on for 30 min. The irradiation sources surrounded the rector and were characterized by a cumulative intensity of 100 W·m^−2^ UV and 4 W·m^−2^ Vis. The temperature of the whole system was stable at 25 °C using the thermostatic chamber. The NO and NO_2_ concentrations were continuously measured in the reactor outlet using a chemiluminescent T200 NOx analyzer (Teledyne, Camino Dos Rios, CA, USA).

## 3. Results and Discussion

### 3.1. Photocatalytic Activity of the Investigated Materials

The new photoactive cements were obtained by the introduction of an intermediate product of TiO_2_ production into cement clinker at different temperatures during its cooling. The modified clinker was ground in a ball mill with 5% gypsum by weight. In the case of cements, gypsum is added during grinding because it affects the cement hydration process because, without the presence of sulfates during hydration, the hardening of cement would take place too quickly, almost immediately after the cement is mixed with water. Since the product taken from the sulfate titanium dioxide production plant (TiO(OH)_2_) also contains sulfates, it was necessary to determine how much sulfur was introduced into the cement when amorphous TiO_2_ was added to it. The sulfur content in unmodified clinker, dried amorphous TiO_2_ and TiO_2_ modified at different temperatures was measured. The results of the tests are presented in Table 2. The sulfur content in the TiO_2_ samples calcined at different temperatures was practically the same, averaging 2% by weight. Since the addition of amorphous TiO_2_ to the clinker during cooling is at levels of 1, 3 and 5 wt.%, it would be possible to reduce the amount of gypsum added in the final clinker grinding process by 2% in the case of using a 1% amorphous TiO_2_ additive and by 5% in the case of cement production with 5 wt.% TiO_2_ content, because the semi-finished product from the TiO_2_ installation using the sulfate method would additionally act as a source of sulfates. In the case of crystallite size measurements, which are also presented in Table 2, there is no relationship between the size of crystallites and the calcination temperature of amorphous TiO_2_. The average size of TiO_2_ crystallites (anatase) is 7–8 nm.

### 3.2. Mechanical Properties of Obtained New Photoactive Cements

**Figure 2:**
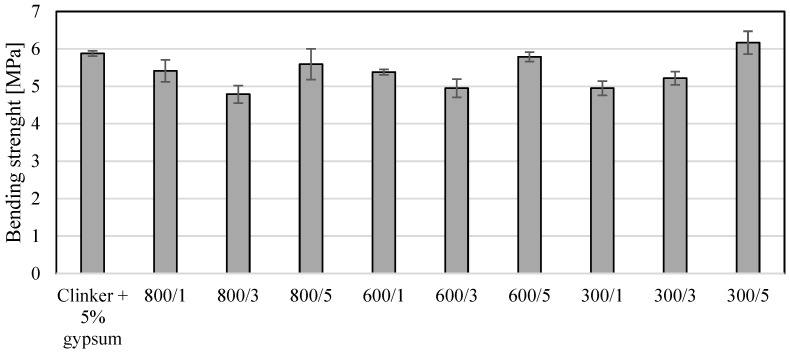
The values of bending strength for modified clinkers.

**Figure 3:**
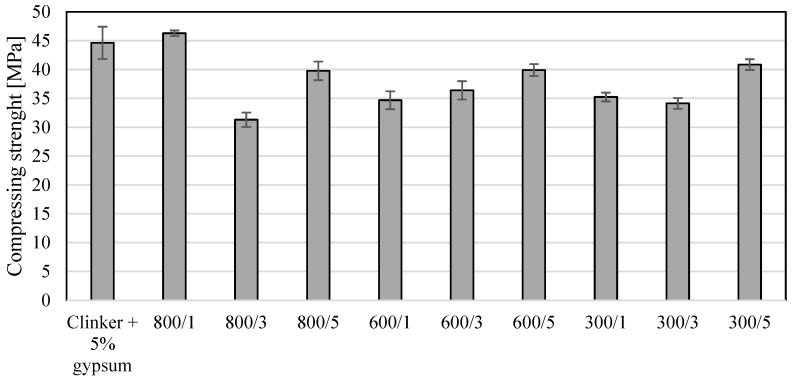
The values of compressing strength for modified clinkers.

Figure 2 shows the bending strength values, and Figure 3 shows the compressive strength values of the modified clinkers. As expected, adding a photocatalyst changed the bending and compressive strength of the modified clinker. As shown in Figure 2 and Figure 3, the addition of TiO_2_ affects the change in the value of compressing strength more than the change in bending strength. Unexpectedly, for the measurement of compressing strength and 5% bending strength, the addition of TiO_2_ had the least effect on changes in these parameters. As shown in Figure 2, the 5% addition of TiO_2_ to clinker at 300, 600 and 800 °C resulted in the lowest changes in bending strength values. When a modification temperature of 800 °C was applied, the bending strength was reduced by 5%, and when a modification temperature of 300 °C was applied, it even resulted in an improvement in bending strength by 5%. In the case of adding 1 and 3 wt.% TiO_2_, regardless of the applied modification temperature, the bending strength was reduced by 10 to even 20%. In the case of the compressing strength (Table 3) of the modified cements, it can be seen that the addition of 1, 3 and 5 wt.% TiO_2_ had a greater effect on the change in this parameter than on bending strength. Nevertheless, as in the case of bending strength, and in the case of compressing strength of 5%, the addition of TiO_2_ reduced the compressive strength to the smallest extent, and it was about 10% regardless of the temperature of the clinker modification. In the case of other temperatures and the addition of TiO_2_ at levels of 1 and 3%, the reduction in compressive strength was 20%, and in one case, the reduction was 30%. In the case of one photoactive cement, the compressing strength was slightly improved at a level of 4%, and it was clinker modified at 800 °C with a 1% weight addition of TiO_2_. For all modified materials, the addition of TiO_2_ in an amount of 1 to 5 wt.% resulted in a decrease in the value of bending strength with the exception of clinker modified at 300 °C with 5wt.% of TiO_2_. Compressing strength also decreased in value in the case of all modified clinkers except clinker modified at 800 °C with a 1% addition of TiO_2_.

**Figure 4:**
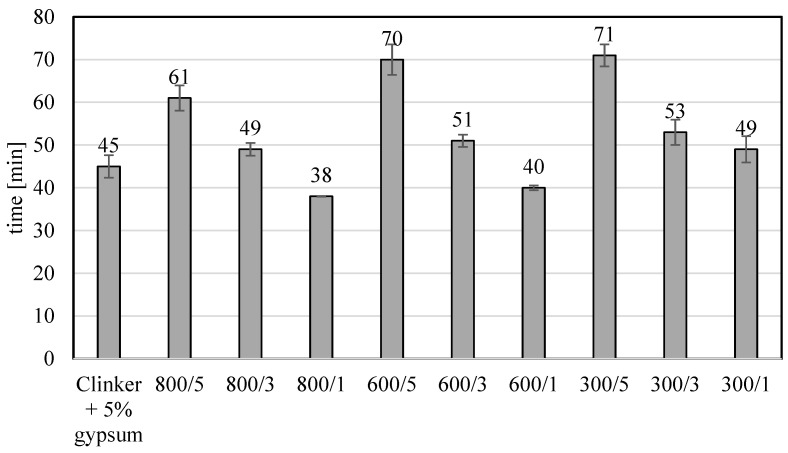
The beginning of the setting time of the modified clinkers.

**Figure 5:**
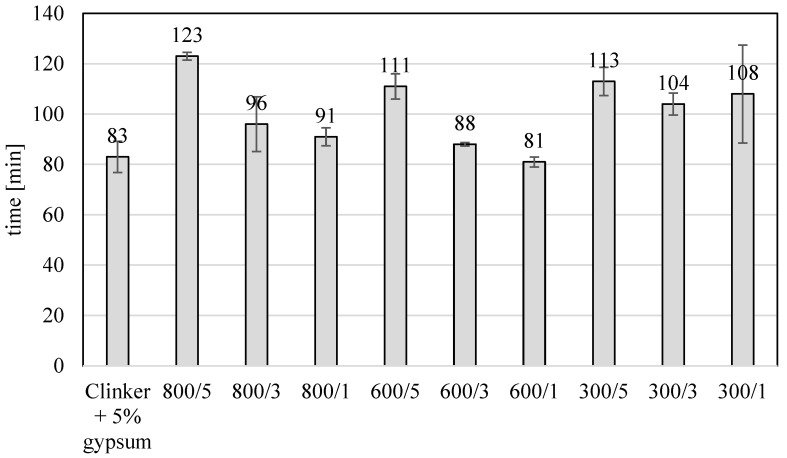
The end of the setting time of the modified clinkers.

The amount of water workability during the construction of the beams was better the higher the percentage of TiO_2_ in the samples. In studies carried out so far, an inverse relationship was observed when the photocatalyst was added to ready-made cements or gypsum. Increasing the amount of photocatalyst reduced the workability of cement and gypsum mortar [14]. Adding a semi-finished product from TiO_2_ installation using the sulfate method to the clinker during its cooling resulted in the clinker being surrounded by a layer of TiO_2_. This resulted in the fact that during grinding, TiO_2_ was in very good contact with the clinker and did not form agglomerates, as observed when adding TiO_2_ to the finished cement. The addition of a larger amount of TiO_2_ improved the workability, so even though TiO_2_ was present in the clinker, with much weaker strength parameters, the improvement in workability compensated for its presence, and the bending strength of materials with 5% TiO_2_ content did not differ significantly from the value for clinker without the addition of TiO_2_. Figure 4 and Figure 5 show the values of the initial and final setting times of modified cement clinkers. According to the literature on the subject, the admixture of TiO_2_ to cements reduces the initial setting time. Wang et al. [15] showed that the addition of 1, 2, 3, 4, and 5 wt.% nano-TiO_2_ reduced the initial setting time by about 40 min from 190 min for cement without the addition of nano-TiO_2_ to about 150 min for cement with the addition of 5 wt.% TiO_2_.

Adding amorphous TiO_2_ in amounts of 3 and 5 wt.% prolongs the initial and final setting time of the modified clinker. With a 5% TiO_2_ content, the initial setting time was increased from 16 to 25 min, compared to clinker without the addition of TiO_2_. A 3% addition of TiO_2_ only slightly increased the setting time, from 4 to 8 min, compared to clinker without TiO_2_. A 1% addition of TiO_2_ slightly reduced the initial setting time. Adding TiO_2_ to the clinker increased the final setting time. As in the case of bending and compressing strength, this behavior should be linked to workability, as it is likely that this increase in workability increased the initial and final setting time. The increase in the initial and final setting times resulted from the presence of 5 wt.% TiO_2_ somewhat prevents the reduction in bending and compressive strength measured after 28 days of curing that would be expected with adding 5 wt.% TiO_2_ to the cement. Like in the case of compressing and bonding strength, adding a semi-finished product from the titanium dioxide installation using the sulfate method to the clinker during its cooling resulted in the clinker being surrounded by a layer of TiO_2_. This resulted in the fact that during grinding, TiO_2_ was in very good contact with the clinker and did not form agglomerates, observed when adding TiO_2_ to the finished cement, which influenced the increase in the time of the beginning and end of setting time.

### 3.3. Photocatalytic Activity Tests

The photocatalytic activity of the new photoactive cements was tested during the decomposition of NO(II) under UV radiation. The initial NO concentration was 1.25 and 0.25 mg·m^−3^. As shown in Table 3, the higher the TiO_2_ content in the cement, the greater the amount of NO that was decomposed. During the tests, no influence of clinker temperature on the degree of NO decomposition was observed. As expected, the lower NO concentration resulted in a greater degree of removal of this gas. The eight modified cement plates with dimensions of 2 × 2 × 0.5 cm used for the study have a specific surface with which the gas is in contact, and at the same time, on this surface, there is a photocatalyst which has a specific number of active sites involved in the NO decomposition reaction. The NO decomposition process occurred continuously, i.e., we had a constant number of active sites available for the gas present in the reactor. As can be seen in Table 3, reducing the NO concentration five times from 1.25 to 0.25 mg·m^−3^ increased the degree of decomposition from 10 to 80%.

The obtained modified cements were tested during operation in five cycles and no changes in their photoactivity were observed.

## 4. Conclusions

In this paper, we prove that it is possible to obtain photoactive cement by modifying the process of obtaining it. Until now, photoactive cements were often obtained by adding photocatalysts to finished cement. Our studies proved that it is possible to use a semi-product from the production of titanium dioxide using the sulfate method and introduce it into a clinker cooler at a temperature of 300 to 800 °C within 30 min. This temperature and the contact time are sufficient for the amorphous structure of TiO_2_ to transform into a crystalline photoactive form. Thanks to this method, it will be possible to reduce the production costs of photoactive cements, because the intermediate product (amorphous TiO_2_) must be obtained from the installation before the calcination process and introduced into the clinker during its cooling. In addition, when mixing clinker at high temperatures with amorphous TiO_2_, the surface of the clinker is coated by amorphous TiO_2_, which undergoes the crystallization process. However, at the same time, it affects the fact that in the next stage of the cement process, i.e., during clinker grinding, the photocatalyst does not agglomerate. It is better dispersed in the cement matrix, which affects the initial and final setting time extended, but there is no drastic reduction in the bending and compressive strength of cements modified with 5% TiO_2_ content by weight.

## 5. Patents

The patent PL237058 protects the new method of obtaining photoactive cements and is currently covered by the European patent application procedure PCT/PL2020/050016.

## Figures and Tables

**Table 1:** Designations of materials adopted in the studies.

Designation	Abbreviation	The Temperature at which the Clinker Was Heated [°C]	Weight % of TiO_2_ to the Weight of Clinker	Weight % of Gypsum to the Weight of Clinker with TiO_2_
clinker + 5% gypsum	clinker + 5% gypsum	-	-	5
clinker-300 °C-TiO_2_-1%	300/1	300	1	5
clinker-300 °C-TiO_2_-3%	300/3	300	3	5
clinker-300 °C-TiO_2_-5%	300/5	300	5	5
clinker-600 °C-TiO_2_-1%	600/1	600	1	5
clinker-600 °C-TiO_2_-3%	600/3	600	3	5
clinker-600 °C-TiO_2_-5%	600/5	600	5	5
clinker-800 °C-TiO_2_-1%	800/1	800	1	5
clinker-800 °C-TiO_2_-3%	800/3	800	3	5
clinker-800 °C-TiO_2_-5%	800/5	800	5	5

**Table 2:** Crystallite size and sulfur content of titanium dioxide obtained at different temperatures.

Material	Sulfur Content [%]	Size of Crystallites [nm]
clinker	1.12	-
Died, amorphous TiO_2_	2.18	7.5
300/1	2.31	8.29
300/3	2.25	7.87
300/5	2.31	8.21
600/1	2.38	7.63
600/3	2.33	7.26
600/5	2.33	6.61
800/1	2.48	7.62
800/3	2.40	7.39
800/5	2.37	7.32

**Table 3:** Photocatalytic decomposition of NO with an initial concentration of 1.25 and 0.25 mg·m^−3^ under UV light irradiation.

Materials	NO Decomposition [%]	
	1.25 mg·m^−3^	0.2 mg·m^−3^
clinker + 5% gypsum	2.2	2.2
300/1	5.5	7
300/3	6.7	7
300/5	6.1	11.5
600/1	5.3	6.1
600/3	9	10.5
600/5	10	11.1
800/1	5.2	9.1
800/3	8.5	10.9
800/5	8.9	12.7

## Data Availability

The original contributions presented in the study are included in the article, further inquiries can be directed to the corresponding author.

## References

[1] Hamidi F., Aslani F. (2019). TiO_2_-based Photocatalytic Cementitious Composites: Materials, Properties, Influential Parameters, and Assessment Techniques. Nanomaterials.

[2] Carmona-Quiroga P.M., Martínez-Ramírez Viles H.A. (2018). Efficiency and durability of a self-cleaning coating on concrete and stones under both natural and artificial ageing trials. Appl. Surf. Sci..

[3] Janus M., Mądraszewski S., Zając K., Kusiak-Nejman E. (2020). A new preparation method of cement with photocatalytic activity. Materials.

[4] Bianchi C.L., Cerrato G., Pirola C., Galli F., Capucci V. (2019). Photocatalytic porcelain grés large slabs digitally coated with AgNPs-TiO_2_. Environ. Sci. Pollut. Res..

[5] Binas V., Papadaki D., Maggos T., Katsanaki A., Kiriakidis A. (2018). Study of innovative photocatalytic cement based coatings: The effect of supporting materials. Const. Build. Mater..

[6] Folli A., Jakobsen U.H., Guerrini G.L., Macphee A. (2009). Rhodamine B Discolouration on TiO_2_ in the Cement Environment: A Look at Fundamental Aspects of the Self-cleaning Effect in Concretes. J. Advanc. Oxid. Technol..

[7] Ratan J.K., Saini A., Verma P. (2018). Microsized-titanium dioxide based self-cleaning cement: Incorporation of calcined dolomite for enhancement of photocatalytic activity. Mater. Res. Exp..

[8] Wang D., Hou P., Zhang L., Xie N., Yang P., Cheng X. (2018). Photocatalytic activities and chemically-bonded mechanism of SiO_2_@TiO_2_ nanocomposites coated cement-based materials. Mater. Res. Bull..

[9] Wang D., Geng Z., Hou P., Yang P., Cheng X., Huang S. (2020). Rhodamine B Removal of TiO_2_@SiO_2_ Core-Shell Nanocomposites Coated to Buildings. Crystals.

[10] Hernández-Rodríguez M.J., Santana Rodríguez R., Darias R., González Díaz O., Pérez Luzardo J.M., Doña Rodríguez J.M., Pulido Melián E. (2019). Effect of TiO_2_ Addition on Mortars: Characterization and Photoactivity. Appl. Sci..

[11] Hewlett P. (2003). Lea’s Chemistry of Cement and Concrete.

[12] (2016). Cement Test Methods—Part 3: Determination of Setting Times and Volume Constancy.

[13] (2016). Cement Test Methods—Part 1: Determination of Strength.

[14] Zając K., Czyżewski A., Kaszyńska M., Janus M. (2020). Combined Effect of Photocatalyst, Superplasticizer and Glass Fiber on the Photocatalytic Activity and Technical Parameters of Gypsum. Catalysts.

[15] Wang L., Zhang H., Gao Y. (2018). Effect of TiO_2_ Nanoparticles on Physical and Mechanical Properties of Cement at Low Temperature. Adv. Mater. Sci. Eng..

